# Supplementary material for: Convergent trends and spatiotemporal patterns of Aedes-borne arboviruses in Mexico and Central America
Source: PLoS Negl Trop Dis. 2023 Sep 6;17(9):e0011169. doi: 10.1371/journal.pntd.0011169 (PMC10506721; doi:10.1371/journal.pntd.0011169)
Supplement: S1 Text — (DOCX) [file pntd.0011169.s001.docx]

**Supplementary Information**

**“Convergent trends and spatiotemporal patterns of Aedes-borne arboviruses in Mexico and Central America”**

**S1 Text: Retrospective on Aedes-borne arbovirus epidemics in the Americas and epidemiological surveillance in Mexico**

*Chikungunya virus (CHIKV)*

The first locally acquired infections were reported in the Caribbean islands in 2013 [1,2], with subsequent spread into Central and South America during 2014 [3–5]. The outbreaks in the Caribbean islands affected various territories over 2014 [6], and reached high incidences in Guadeloupe (295.8 cases per 1000 inhabitants in November 2014), the French Guiana (112.41 cases per 1000 inhabitants in July 2014) and the Dominican Republic (over 142 000 new cases reported in July 2014). CHIKV reached South America and the USA by June 2014, followed by Mexico and various other countries in Central America by the end of that year [7]. Most of these outbreaks have been attributed to a single introduction of the Asian genotype into the Eastern Caribbean [8], except for Brazil, where a secondary and independent introduction event of the Eastern/Central/South African (ECSA) virus genotype was identified [9]. The ECSA genotype has since then spread across Brazil [10,11], and was recently introduced into Paraguay [12]. It is believed that the spread of CHIKV in the region was at least partially driven by international travel [13–15], whilst a successful virus establishment was likely aided by (*i*) the lack of prior immunity to the virus in the Americas, (*ii*) the presence of both *Ae. aegypti* and *Ae. albopictus* in the same geographical region, and (*iii*) and elevated volumes of travel across countries with high virus transmission [16]. CHIKV was detected in Mexico in 2014, with the highest number of cases reported during 2015, particularly within the southern and southwestern states of Guerrero, Michoacán and Yucatán [17]. From 2016 onwards, limited numbers of cases have been reported, although CHIKV incidence in southwestern states (such as Guerrero) is thought to be underestimated, represented by undiagnosed or non-hospitalized cases that do not make it to the SINAVE official case report list [18].

*Dengue virus (DENV)*

DENV is classified into four immunologically and genetically distinct serotypes: DENV-1, DENV-2, DENV-3 and DENV-4. Before the 1960s, DENV-2 was the only serotype detected in the Americas. Nonetheless, the other three serotypes were detected throughout the 1960s to the 1990s, including the characterization of multiple introductions of genetically distinct DENV-2 lineages [19,20]. Once established within a geographical region, DENV epidemics follow seasonal patterns (as observed in countries in the Americas) [21–25], with prominence in regions where the virus has become endemic (defined as where at least one virus serotype constantly circulates [26]) or hyperendemic (defined as where multiple virus serotypes constantly co-circulating). In these scenario, seasonal cycles are characterised by yearly periods where outbreaks are more frequent, driven in part by changing climatic conditions that favour vector breeding and increased vector-host interactions [27,28]. Furthermore, the co-circulation of different DENV serotypes can lead to dominance over specific season across multiple years [29–31]. The processes driving t long-term dominance patterns are complex and involve climate, vector ecology, demographics and immunity of the host population factors [32–34]. This can lead to seasonal patterns where large outbreaks vary in size and severity (for example, with higher number of cases for dengue haemorrhagic fever, DHF) across seasons through time [21–25].

Dengue has been under surveillance in Mexico since the 1980s and became a health concern at a national level when the incidence of DHF increased during the 1990s, associated with the spread of *Aedes* mosquito populations in Mexico [35]. Since the 2000s, the geographical distribution of Dengue has marginally expanded into other locations closer to central Mexico, such as the state of Morelos and areas of higher altitude, like the state of Guerrero [36,37]. During the early 2010s, epidemiological hotspots for Dengue were identified in and around urban areas within the southern states, where transmission occurs year-round but peaks during the rainy season [37]. The incidence of DENV serotypes has fluctuated over multiple years: during the early 2000s, DENV-1 and DENV-2 alternated as the dominant serotype in Mexico, with DENV-1 replacing the previously dominant DENV-2 between 2004 and 2007 [36].

**References**

1. Lanciotti RS, Valadere AM. Transcontinental movement of Asian Genotype Chikungunya virus. Emerg Infect Dis. 2014;20: 1400–1402. doi:10.3201/eid2008.140268

2. Nasci RS. Movement of Chikungunya virus into the Western Hemisphere. Emerg Infect Dis. 2014;20: 1394–1395. doi:10.3201/eid2008.140333

3. Cunha MS, Costa PAG, Correa IA, de Souza MRM, Calil PT, da Silva GPD, et al. Chikungunya Virus: An Emergent Arbovirus to the South American Continent and a Continuous Threat to the World. Front Microbiol. 2020;11. doi:10.3389/fmicb.2020.01297

4. Berry IM, Rutvisuttinunt W, Sippy R, Beltran-ayala E, Figueroa K, Ryan S, et al. The origins of dengue and chikungunya viruses in Ecuador following increased migration from Venezuela and Colombia. 2020;8: 1–12.

5. Camacho D, Reyes J, Negredo A, Hernández L, Sánchez-Seco M, Comach G. Asian genotype of Chikungunya virus circulating in Venezuela during 2014. Acta Trop. 2017;174: 88–90. doi:10.1016/j.actatropica.2017.06.026

6. Mowatt L, Jackson ST. Chikungunya in the Caribbean: An Epidemic in the Making. Infect Dis Ther. 2014;3: 63–68. doi:10.1007/s40121-014-0043-9

7. Johansson MA. Chikungunya on the move. Trends Parasitol. 2015;31: 43–45. doi:10.1016/j.pt.2014.12.008

8. Leparc-Goffart I, Nougairede A, Cassadou S, Prat C, De Lamballerie X. Chikungunya in the Americas. The Lancet. 2014;383: 514. doi:10.1016/S0140-6736(14)60185-9

9. Nunes MRT, Faria NR, de Vasconcelos JM, Golding N, Kraemer MUG, de Oliveira LF, et al. Emergence and potential for spread of Chikungunya virus in Brazil. BMC Med. 2015;13. doi:10.1186/s12916-015-0348-x

10. Naveca FG, Claro I, Giovanetti M, de Jesus JG, Xavier J, Iani FC de M, et al. Genomic, epidemiological and digital surveillance of Chikungunya virus in the Brazilian Amazon. PLoS Negl Trop Dis. 2018;13: 1–21. doi:10.1371/journal.pntd.0007065

11. Xavier J, Giovanetti M, Fonseca V, Thézé J, Gräf T, Fabri A, et al. Circulation of chikungunya virus East/Central/ South African lineage in Rio de Janeiro, Brazil. PLoS One. 2019;14: 1–14. doi:10.1371/journal.pone.0217871

12. de Oliveira EC, Fonseca V, Xavier J, Adelino T, Claro IM, Fabri A, et al. Short report: Introduction of chikungunya virus ecsa genotype into the brazilian midwest and its dispersion through the americas. PLoS Negl Trop Dis. 2021;15: 1–10. doi:10.1371/journal.pntd.0009290

13. Johansson MA, Powers AM, Pesik N, Cohen NJ, Erin Staples J. Nowcasting the spread of Chikungunya Virus in the Americas. PLoS One. 2014;9. doi:10.1371/journal.pone.0104915

14. Khan K, Bogoch I, Brownstein JS, Miniota J, Nicolucci A, Hu W, et al. Assessing the Origin of and Potential for International Spread of Chikungunya Virus from the Caribbean. PLoS Curr. 2014; 4055609. doi:10.1371/currents.outbreaks.2134a0a7bf37fd8d388181539fea2da5

15. Escobar LE, Qiao H, Peterson AT. Forecasting Chikungunya spread in the Americas via data-driven empirical approaches. Parasit Vectors. 2016;9: 1–12. doi:10.1186/s13071-016-1403-y

16. Weaver SC. Arrival of Chikungunya Virus in the New World: Prospects for Spread and Impact on Public Health. PLoS Negl Trop Dis. 2014;8: 6–9. doi:10.1371/journal.pntd.0002921

17. Nava-Frías M, Searcy-Pavía RE, Juárez-Contreras CA, Valencia-Bautista A. Chikungunya fever: Current status in Mexico. Bol Med Hosp Infant Mex. 2016;73: 67–74. doi:10.1016/j.bmhimx.2016.03.001

18. Nunez-Avellaneda D, Tangudu C, Barrios-Palacios J, Salazar MaI, Machain-Williams C, Cisneros-Pano J, et al. Chikungunya in Guerrero, Mexico, 2019 and Evidence of Gross Underreporting in the Region. Am J Trop Med Hyg. 2021;105: 1281–1284. doi:10.4269/ajtmh.21-0431

19. Pinheiro F, Nelson M. Re-Emergence of Dengue and Emergence of Dengue Haemorrhagic Fever in the Americas. Dengue Bull. 1997;21: 1–6.

20. Allicock OM, Lemey P, Tatem AJ, Pybus OG, Bennett SN, Mueller BA, et al. Phylogeography and population dynamics of dengue viruses in the Americas. Mol Biol Evol. 2012;29: 1533–1543. doi:10.1093/molbev/msr320

21. Gordon A, Kuan G, Mercado JC, Gresh L, Avile W, Balmaseda A, et al. The Nicaraguan Pediatric Dengue Cohort Study : Incidence of Inapparent and Symptomatic Dengue Virus. 2013;7: 2004–2010. doi:10.1371/journal.pntd.0002462

22. Tami A, Lizarazo EF, Grillet ME. ENSO-driven climate variability promotes periodic major outbreaks of dengue in Venezuela. Sci Rep. 2018; 1–11. doi:10.1038/s41598-018-24003-z

23. Id RS, Herrera D, Gaus D, Gangnon RE, Patz A, Id JEO. Seasonal patterns of dengue fever in rural Ecuador : 2009-2016. 2019; 2009–2016.

24. Churakov M, Villabona-Arenas CJ, Kraemer MUG, Salje H, Cauchemez S. Spatio-temporal dynamics of dengue in Brazil: Seasonal travelling waves and determinants of regional synchrony. PLoS Negl Trop Dis. 2019;13: 1–13. doi:10.1371/journal.pntd.0007012

25. Balmaseda A, Standish K, Mercado JC, Matute JC, Tellez Y, Saborío S, et al. Trends in patterns of dengue transmission over four years of a pediatric cohort study in Nicaragua. J Infect Dis. 2010;201: 5–14. doi:10.1086/648592.Trends

26. Guo RN, Lin JY, Li LH, Ke CW, He JF, Zhong HJ, et al. The prevalence and endemic nature of dengue infections in Guangdong, South China: An epidemiological, serological, and etiological study from 2005-2011. PLoS One. 2014;9. doi:10.1371/journal.pone.0085596

27. Halstead SB. Dengue virus-mosquito interactions. Annu Rev Entomol. 2008;53: 273–291. doi:10.1146/annurev.ento.53.103106.093326

28. Stewart-ibarra AM, Lowe R. Climate and Non-Climate Drivers of Dengue Epidemics in Southern Coastal Ecuador. 2013;88: 971–981. doi:10.4269/ajtmh.12-0478

29. Adams B, Holmes EC, Zhang C, Mammen MP, Nimmannitya S, Kalayanarooj S, et al. Cross-protective immunity can account for the alternating epidemic pattern of dengue virus serotypes circulating in Bangkok. 2006.

30. Zhang C, Mammen MP, Chinnawirotpisan P, Klungthong C, Rodpradit P, Monkongdee P, et al. Clade Replacements in Dengue Virus Serotypes 1 and 3 Are Associated with Changing Serotype Prevalence †. 2005;79: 15123–15130. doi:10.1128/JVI.79.24.15123

31. Zhang Q, Chen Y, Fu Y, Liu T, Zhang Q, Guo P. Epidemiology of dengue and the effect of seasonal climate variation on its dynamics : a spatio-temporal descriptive analysis in the Chao-Shan area on China ’ s southeastern coast. 2019. doi:10.1136/bmjopen-2018-024197

32. Ferguson N, Anderson R, Gupta S. The effect of antibody-dependent enhancement on the transmission dynamics and persistence of multiple-strain pathogens. Proc Natl Acad Sci U S A. 1999;96: 790–794. doi:10.1073/pnas.96.2.790

33. Recker M, Blyuss KB, Simmons CP, Hien TT, Wills B, Farrar J, et al. Immunological serotype interactions and their effect on the epidemiological pattern of dengue. Proceedings of the Royal Society B: Biological Sciences. 2009;276: 2541–2548. doi:10.1098/rspb.2009.0331

34. Wagner CE, Hooshyar M, Baker RE, Yang W, Arinaminpathy N, Vecchi G, et al. Climatological , virological and sociological drivers of current and projected dengue fever outbreak dynamics in Sri Lanka. 2020.

35. Montesano-Castellanos R, Ruiz-Matus C. Vigilancia epidemiológica del dengue en México. Salud Publica Mex. 1995;370: S64–S76.

36. Dantés HG, Farfán-Ale JA, Sarti E. Epidemiological Trends of Dengue Disease in Mexico (2000–2011): A Systematic Literature Search and Analysis. PLoS Negl Trop Dis. 2014;8. doi:10.1371/journal.pntd.0003158

37. Dzul-Manzanilla F, Correa-Morales F, Che-Mendoza A, Palacio-Vargas J, Sánchez-Tejeda G, González-Roldan JF, et al. Identifying urban hotspots of dengue, chikungunya, and Zika transmission in Mexico to support risk stratification efforts: a spatial analysis. Lancet Planet Health. 2021;5: e277–e285. doi:10.1016/S2542-5196(21)00030-9
