# Supplementary figures and images for: Convergent trends and spatiotemporal patterns of Aedes-borne arboviruses in Mexico and Central America
Source: PLoS Negl Trop Dis. 2023 Sep 6;17(9):e0011169. doi: 10.1371/journal.pntd.0011169 (PMC10506721; doi:10.1371/journal.pntd.0011169)

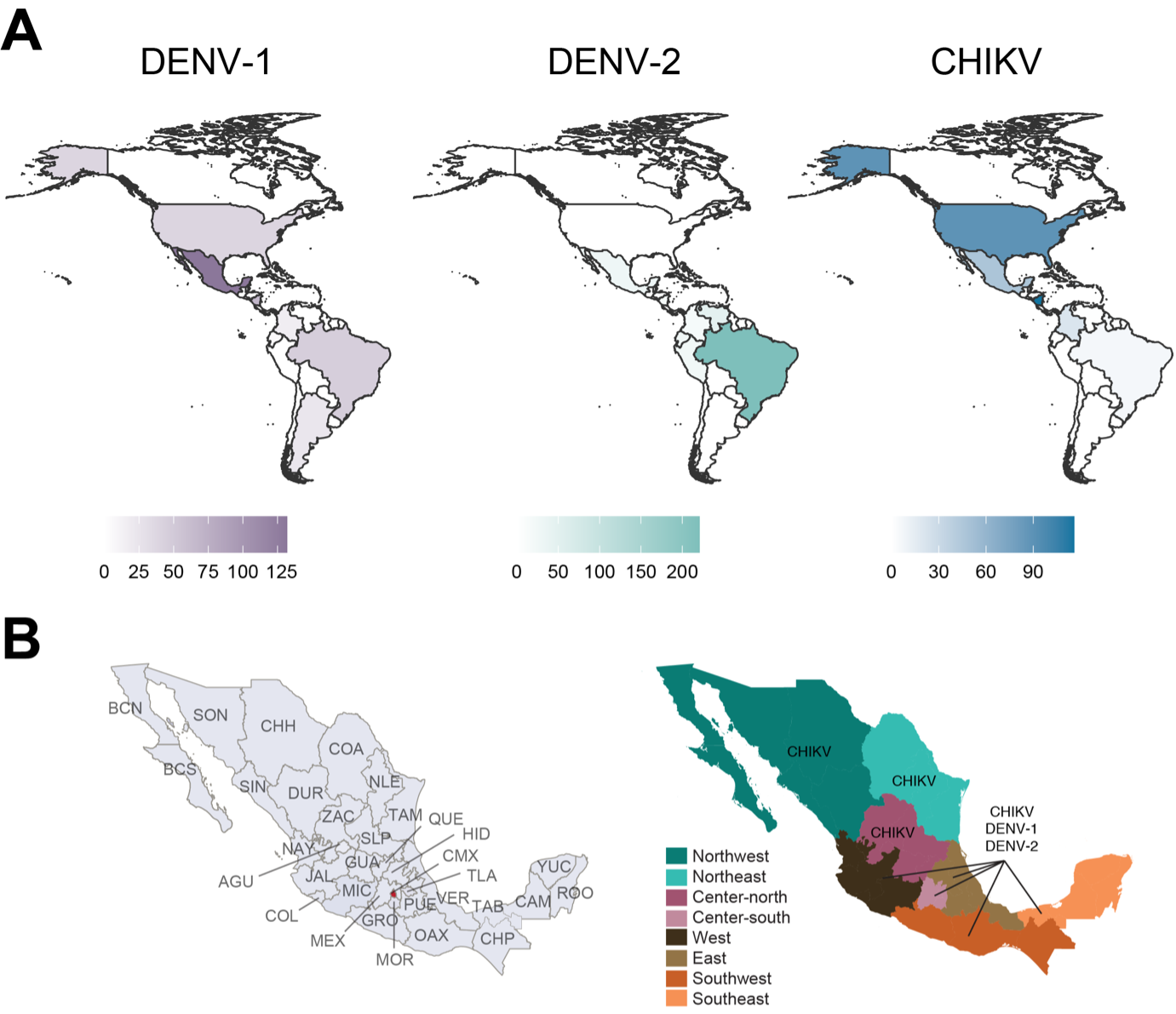

Supplement: S1 Fig — (A) Geographic distribution of DENV-1, DENV-2 and CHIKV virus genomes from the region of the Americas included for phylogeographic analyses in this study. Maps are coloured according to the number of sequences sampled per country. (B) Geographic distribution of DENV-1, DENV-2 and CHIKV virus genomes from Mexico included in this study. The map on left indicates the 32 states from the country, whilst the map of the right shows in different colours the distinct geographic regions (comprising different states) from which Aedes-borne virus genomes are available from. Plots were generated using the ggplot package (https://ggplot2.tidyverse.org/index.html) in R. Original base layer maps use as a source for geospatial base layer data public domain maps imported from the Natural Earth data project (https://cran.r-project.org/web/packages/rnaturalearth/index.html). (PNG) [file pntd.0011169.s002.png]

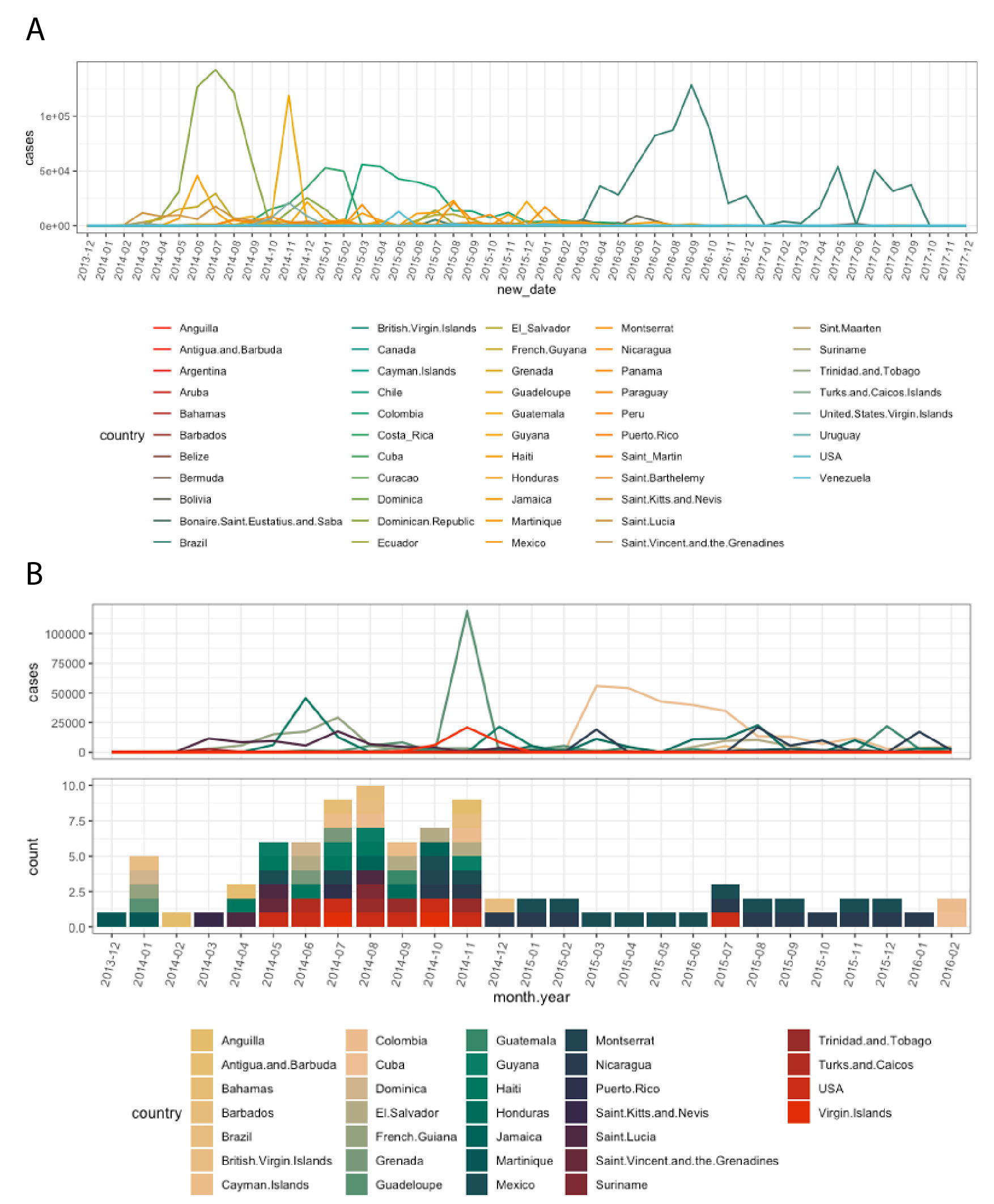

Supplement: S2 Fig — (A) Monthly number of CHIKV cases reported to the Pan-American Health Organisation (PAHO) between 2013 and 2017, grouped by country. (B) Comparison between monthly number of cases (reported to PAHO, upper panel) in countries that have generated CHIKV genome sequences, and publicly available complete CHIKV genome sequences (lower panel). Mexico sequences include those generated in this study. Plots were generated using the ggplot package (https://ggplot2.tidyverse.org/index.html) in R. (PNG) [file pntd.0011169.s003.png]

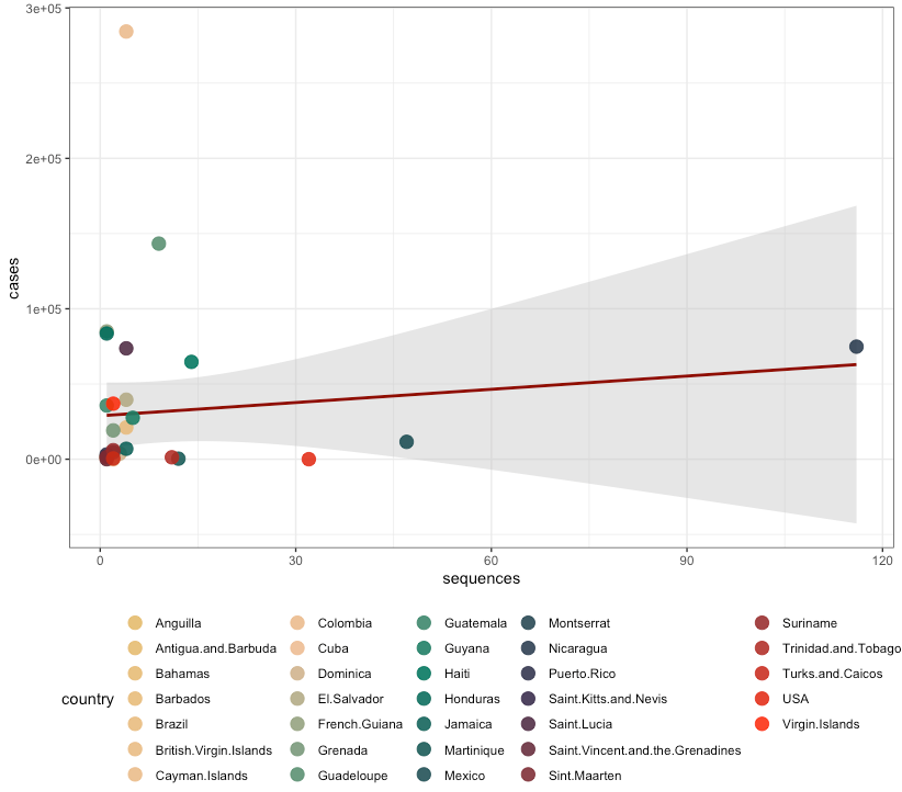

Supplement: S3 Fig — A Spearman’s Rho = 0.26, p = 0.15 denotes no correlation between the cumulative number of cases per country versus the number of viral genome sequences available per country. Plots were generated using the ggplot package (https://ggplot2.tidyverse.org/index.html) in R. (PNG) [file pntd.0011169.s004.png]

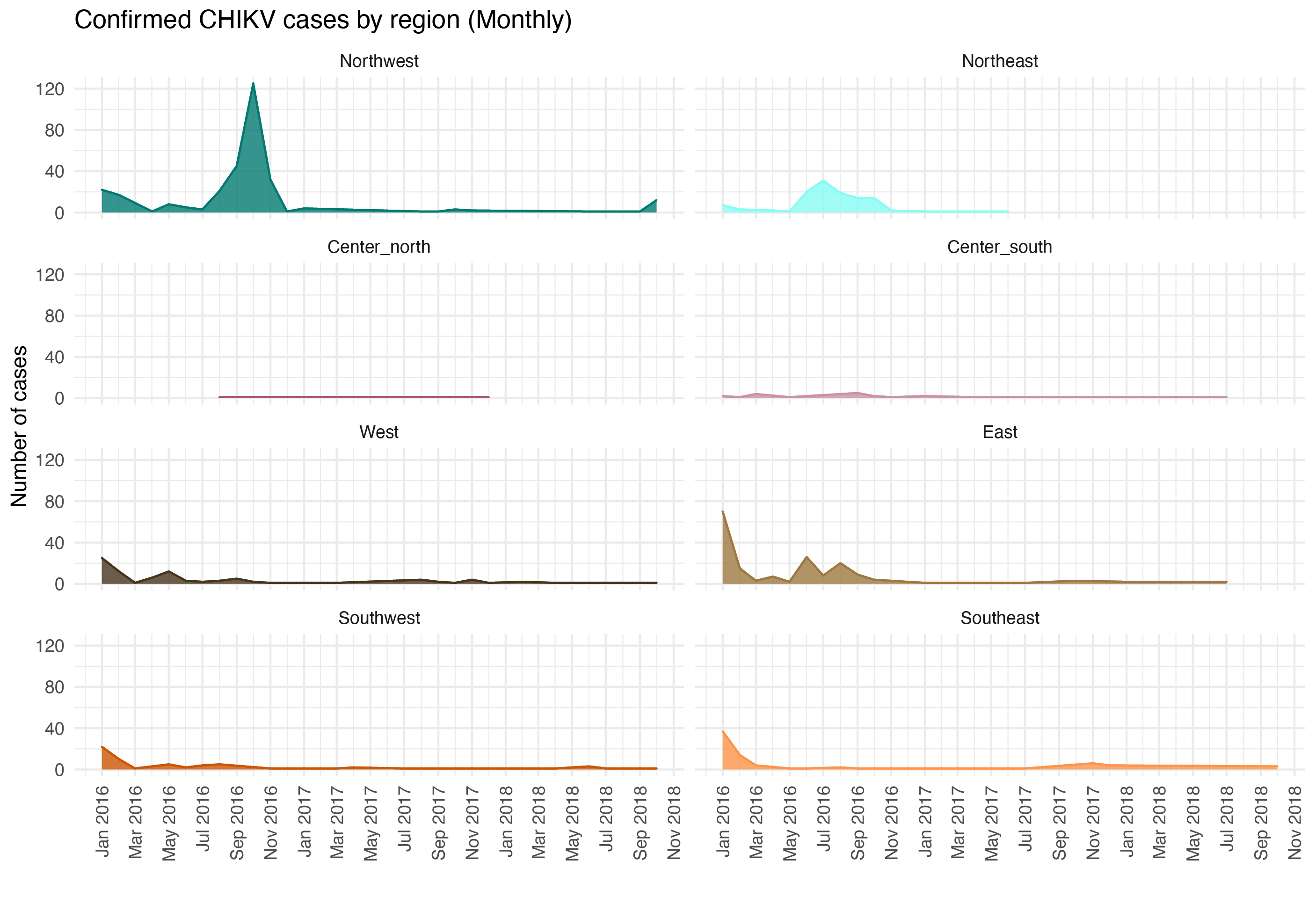

Supplement: S4 Fig — Monthly cases for CHIKV reported under the SINAVE surveillance system (InDRE/Ministry of Health Mexico). Plots were generated using the ggplot package (https://ggplot2.tidyverse.org/index.html) in R. (PNG) [file pntd.0011169.s005.png]

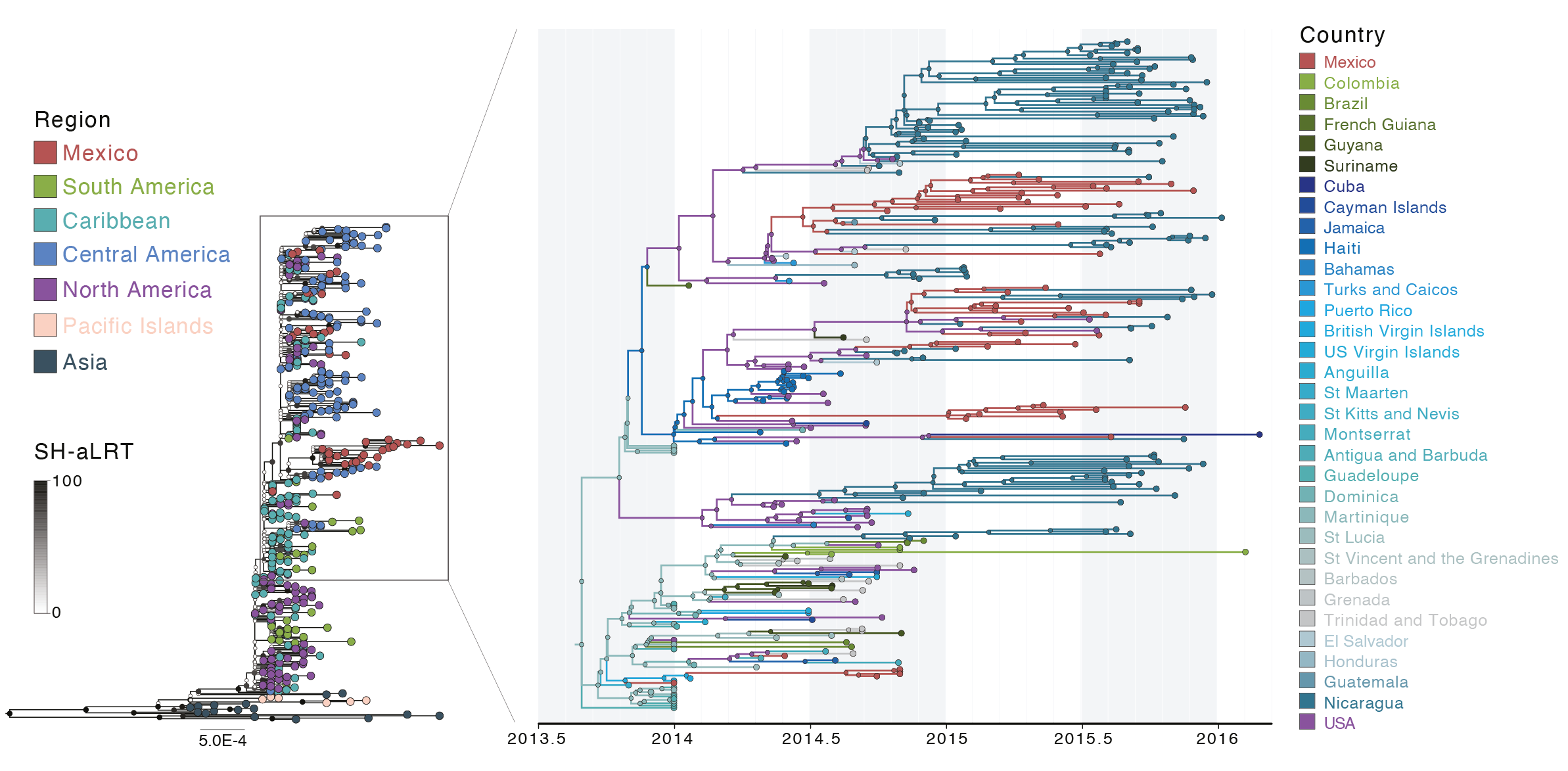

Supplement: S5 Fig — ML phylogenetic tree for CHIKV inferred from the complete genome sequences from the Americas included in our analysis, denoting an ‘American’ lineage (left panel). Tree tips are coloured according to the region/country of collection, whilst nodes are coloured according to branch support values (SH-aLRT). To the right, a time-calibrated MCC tree is displayed, showing a well-defined CCNA clade within the ‘American’ lineage. Tips and branches are coloured by the location of origin and circulation, inferred through a DTA phylogeographic analysis (see Methods section, main text). (PNG) [file pntd.0011169.s006.png]

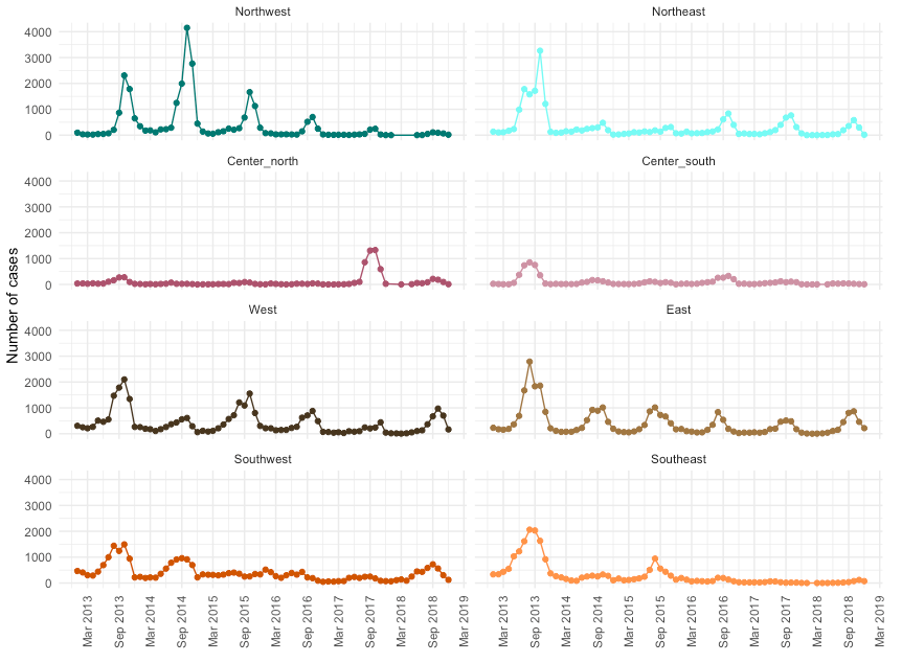

Supplement: S6 Fig — Monthly cases for DENV (aggregating both dengue fever and dengue haemorrhagic fever) reported under the SINAVE surveillance system (InDRE/Ministry of Health Mexico). Plots were generated using the ggplot package (https://ggplot2.tidyverse.org/index.html) in R. (PNG) [file pntd.0011169.s007.png]

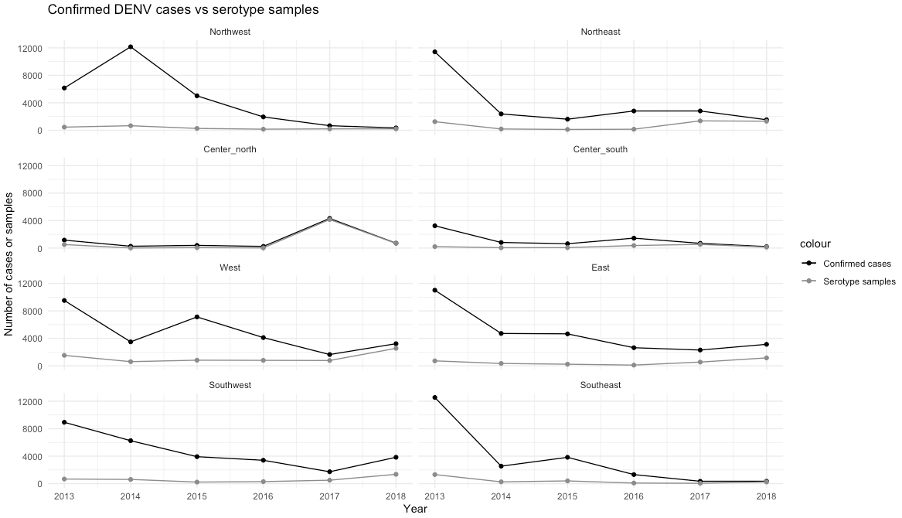

Supplement: S7 Fig — Total number of DENV cases reported by year (black line), compared to total number of DENV cases where the causal serotype has been identified and reported (grey line). Data corresponds to the period of time between 2013 and 2018, as reported under the SINAVE surveillance system (InDRE/Ministry of Health Mexico). Plots were generated using the ggplot package (https://ggplot2.tidyverse.org/index.html) in R. (PNG) [file pntd.0011169.s008.png]

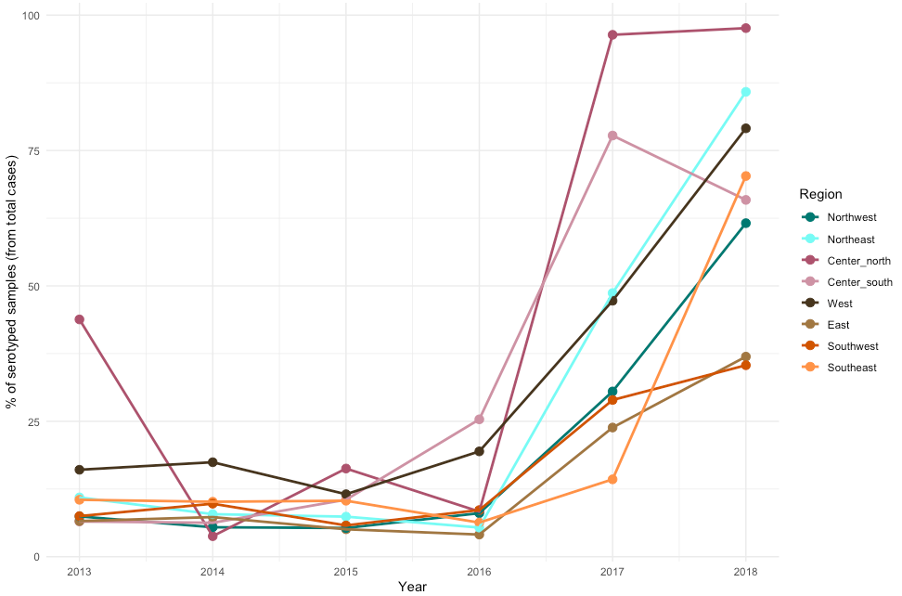

Supplement: S8 Fig — Percentage of serotyped samples per region per year between 2013 and 2018. Plots were generated using the ggplot package (https://ggplot2.tidyverse.org/index.html) in R. (PNG) [file pntd.0011169.s009.png]

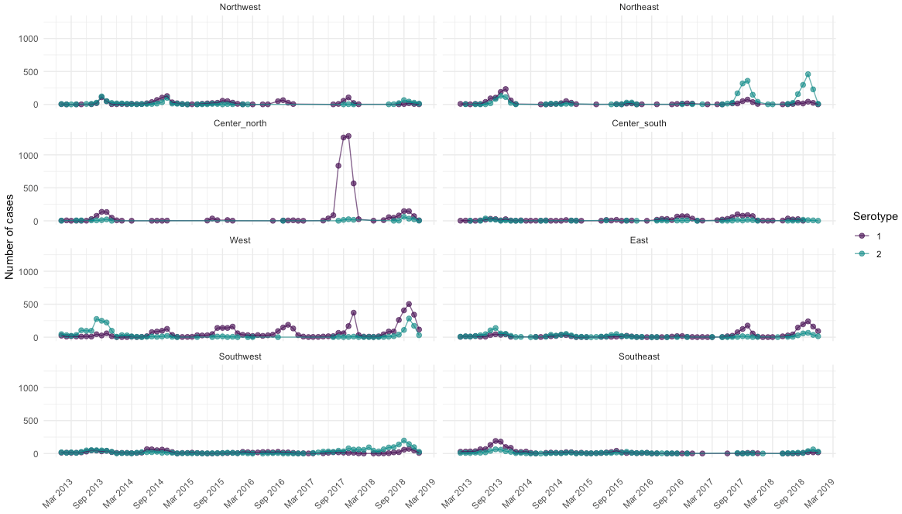

Supplement: S9 Fig — Monthly numbers of cases identified as DENV-1 (purple) or DENV-2 (teal) between 2013 and 2018 across geographic regions in the country. Plots were generated using the ggplot package (https://ggplot2.tidyverse.org/index.html) in R. (PNG) [file pntd.0011169.s010.png]

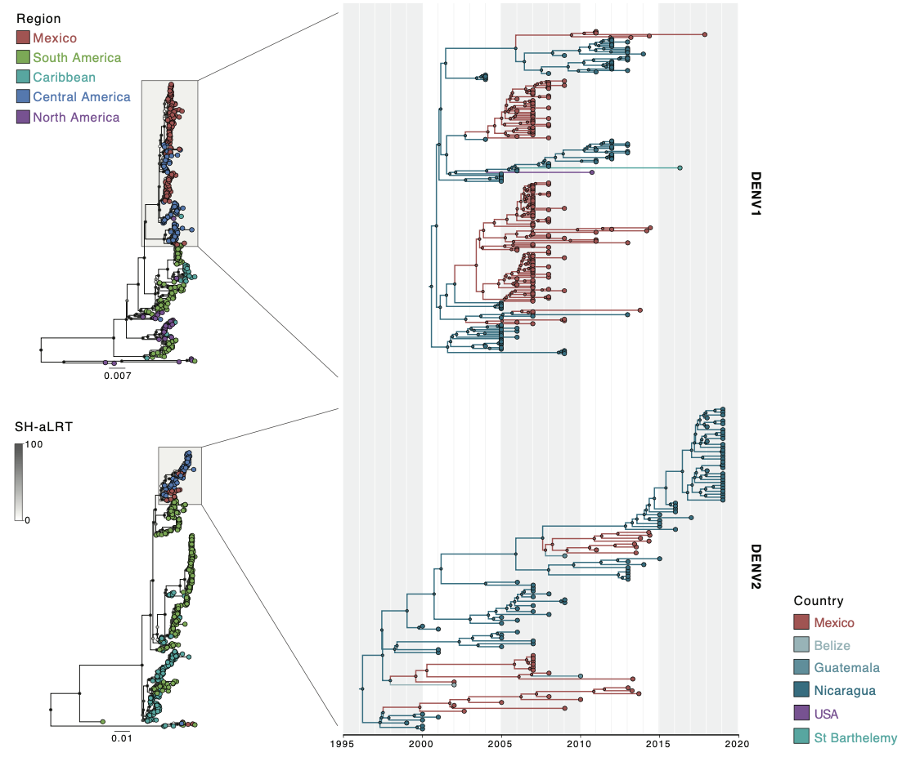

Supplement: S10 Fig — ML phylogenetic trees for DENV-1 (above) and for DENV-2 (below) inferred from the complete genome sequences from the Americas included in our analysis are shown to the left. Tree tips are coloured according to the country/region of collection, whilst nodes are coloured according to branch support values (SH-aLRT). To the right, the time-calibrated MCC trees are displayed, showing well-defined CCNA clades for each virus. Tips and branches are coloured by the location of origin and circulation, inferred through a DTA phylogeographic analysis (see Methods section, main text). (PNG) [file pntd.0011169.s011.png]

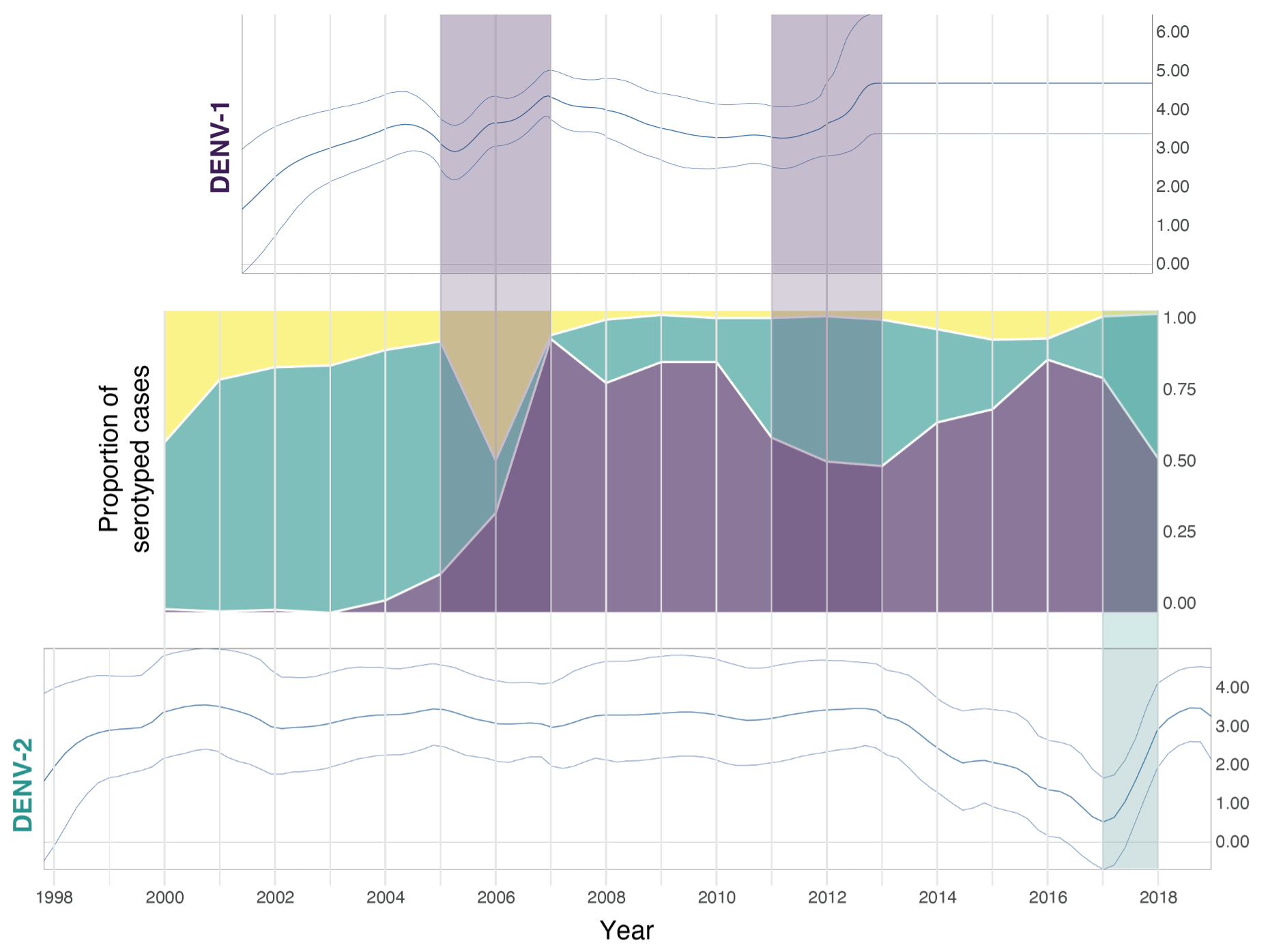

Supplement: S11 Fig — Upper and lower panels show the Bayesian Skyline plots (BSPs) obtained from the time-scaled phylogenetic analyses of the DENV-1 and DENV-2 CCNA lineages. The middle panel shows the proportion of serotyped cases for each DENV serotype in Mexico over a comparable period of time. Shading in purple (for DENV-1) and green (for DENV-2) show periods of time where an increase in the virus effective population size over time was observed (as suggested by the BSP), highlighting the proportion of virus serotypes. (PNG) [file pntd.0011169.s012.png]
