# Supplementary material for: Convergent trends and spatiotemporal patterns of Aedes-borne arboviruses in Mexico and Central America
Source: PLoS Negl Trop Dis. 2023 Sep 6;17(9):e0011169. doi: 10.1371/journal.pntd.0011169 (PMC10506721; doi:10.1371/journal.pntd.0011169)
Supplement: Attachment — Submitted filename: Response_PNTD-D-23-00195.pdf [file pntd.0011169.s021.pdf]

Revised Submission
PNTD-D-23-00195
"Convergent trends and spatiotemporal patterns of arboviruses in Mexico and Central America"
Academic Editor: Aparna Krishnavajhala, Elvina Viennet

General response from authors:

We express our gratitude to the editors and reviewers for their positive feedback and constructive comments. To enhance the clarity and readability of our study, we have incorporated the suggested changes. Below, we provide a detailed response addressing each raised issue, with line numbers corresponding to the tracked version of the manuscript.

We believe that our manuscript is now prepared for publication and kindly request your consideration.

Please feel free to contact me should you have any additional inquiries.

Yours faithfully,
Marina Escalera Zamudio, Ph.D.
Leverhulme Trust ECR fellow
Department of Biology
University of Oxford
Oxford, United Kingdom
marina.escalerazamudio@biology.ox.ac.uk

RESPONSE TO REVIEWERS

1. **Methods**

Reviewer #1: The objectives of the study are clearly stated, with appropriate study samples selected. More information on the selection process (if any are used) for samples sent to InDRE for long-term storage etc. would be useful and help to further contextualise the study samples. The methods used by the authors for CHIKV and DENV sequence generation, investigating epidemiological trends and phylogenetic and phytogeographic analyses are standard yet appropriate given the objectives, nature of the two viruses, as well as the scale of the study.

Reviewer #2 and 3 had no further suggestions

*We thank the Reviewer for raising this remark. We now provide further information on the sample selection process for sending these to InDRE for long-term storage. This is now available as a new paragraph in the introduction section corresponding to **L56-84**:*

"The SINAVE system involves nationwide case reporting by hospitals and clinics within and outside Mexico's Ministry of Health's healthcare network. Employing a sentinel approach based on positive case definition, healthcare institutions select and ship samples (along with essential information such as patient demographics, symptoms, and initial laboratory test results) to designated State Public Health Laboratories (LESP) for diagnosis confirmation using reference cellular and molecular biology standards. Subsequently, positive samples are sent from the LESP network to InDRE (Instituto de Diagnóstico y Referencia Epidemiológicos Dr. Manuel Martínez Báez, Ministry of Health of Mexico, Mexico City) for long-term storage. This enables a centralized historical collection spanning multiple decades and locations, providing a longitudinal perspective for the surveillance of infectious diseases in Mexico. The General Directorate of Epidemiology (Dirección General de Epidemiología) oversees SINAVE in centralizing and compiling data on confirmed cases reported nationwide, in order to facilitate the development of outbreak response measures and the dissemination of epidemiological information. Specifically, for Aedes-borne virus surveillance, blood and serum samples are typically shipped to the National Arbovirus Reference Laboratory, a PAHO-WHO collaborating centre at InDRE [26]."

2. **Results**

Reviewer #2: While the results are presented clearly and completely there are several improvements to suggest. In the Methods sub-section (lines 119-121) describing phylogenetic analysis, there is a reference to the genomic dataset compiled for the analysis, which is provided as Supplementary Data 4. For better understanding of the geographical context and study results it would be good to include as a separate figure a map showing locations where the viruses were isolated.

*We have included additional information regarding the geographical context of the sequences used in our analyses, including those generated in this study. This information can be found in the **Supplementary Information, Figure S1 "Geographic distribution of Aedes-borne arbovirus genome sequences sampled for phylogeographic analyses"**, Additionally, the paragraph corresponding to **L185-204** has been revised accordingly: "Our final datasets include a total of 420 CHIKV, 375 DENV-1, and 643 DENV-2 genome sequences, selected to represent all countries in North and Central America with virus genome representation sampled though time. Datasets encompassed both sequences generated in our study and those obtained from public databases (accession numbers available in*

Supplementary Data 4*). The geographical distribution of the sequences sampled for all analyses can be found in the* ***Supplementary Information, Fig S1."***

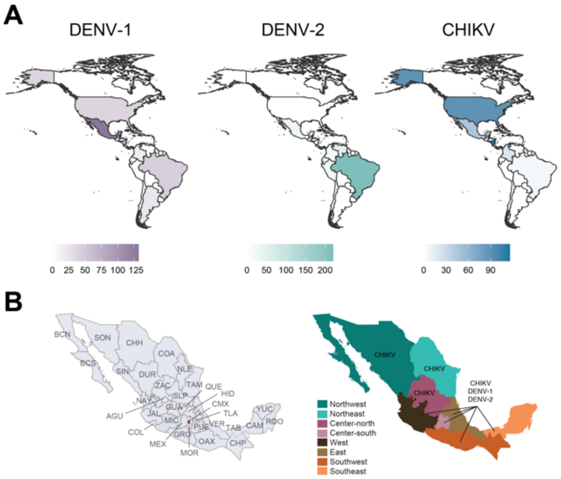

(A) Geographic distribution of DENV-1, DENV-2 and CHIKV virus genomes from the region of the Americas included for phylogeographic analyses in this study. Maps are coloured according to the number of sequences sampled per country. ***(B)*** Geographic distribution of DENV-1, DENV-2 and CHIKV virus genomes from Mexico included in this study. The map on left indicates the 32 states from the country, whilst the map of the right shows in different colours the distinct geographic regions (comprising different states) from which Aedes-borne virus genomes are available from.

The reference to Table 1 in the Methods section (line 157-158) seem to be an error, the mention of the table in the Results seem correct.

We have amended this accordingly

Reviewer #1 and 3 had no further suggestions

3. Conclusions

Reviewer #1: The limitations of the methodology and analyses, in particular the phylogeographic analyses, are clearly outlined and accounted for within the Discussion section of the manuscript. The main findings are adequately compared with those from previous work and other work done in the region. Further discussion of the comparison of the findings, trends etc. identified for CHIKV and DENV in Mexico would strengthen the Discussion section.

*Following the suggestion made, we now provide an extended paragraph discussing the epidemiological trends identified for CHIKV and DENV in Mexico (**L781-807**):*

"Despite apparent contrasting epidemiological trends observed among emerging and established Aedes-borne arboviruses (e.g. DENV vs CHIKV), it is expected that emerging viruses, at some point, will display seasonal patterns coupled with a prolonged regional endemicity [76]. This is clear in countries like India and Thailand, where CHIKV has circulated for an extended period of time. In such regions, cases tend to peak primarily during the rainy season, given the favourable environmental conditions that contribute to the breeding and proliferation of Aedes mosquitoes [77,78]. In the Americas, a similar pattern were already observed during the initial CHIKV outbreak in the Caribbean [76]. Subsequently, in Brazil, where the CHIKV has circulated for longer, seasonality is evident, with an increased virus transmission over time occurring from December to March in most parts of the country [51,79]. In Mexico, only a handful of new CHIKV cases have been officially reported after 2018 [80]. However, serological surveys across different southern states denote a significant disease incidence and prevalence [81], suggesting that there is substantial underreporting of cases nationwide. Thus, we speculate that in Mexico, CHIKV is likely to circulate cryptically, where a prolonged virus endemicity could exhibit seasonal epidemiological trends (similar to those historically observed for DENV). However, only an increased targeted surveillance in high-risk states could shed light on the cryptic epidemiological behaviour of CHIKV, as well as other emerging and established Aedes-borne arboviruses circulating in Mexico."

Reviewer #2 and 3 had no further suggestions

We have amended this accordingly

4. Editorial and Data Presentation Modifications

Reviewer #1: A few minor grammatical errors to be corrected throughout the text.

We have now revised the text and amended all grammatical errors accordingly.

Reviewer #2: Some of the literature sources cited in the texts don't seem to be appropriate as they are not about arboviruses: i.e. references 2, 15 (Lassa fever virus), 3 (MERSV), 9 (SARS-COV2), 16 (Rabies virus), 21 (Ebola virus). More appropriate sources pertaining to arboviruses and supporting the statements exist in the literature. References 36-39 and 40-42, 83, 84 appear in the text not in the sequential order. 3) References 94-111 are not present in the main text. It looks like they are cited in Supplementary Text 1 but the supplementary should have its own list of references. According to the journals' rules literature citations should be in square brackets in the text. References 74, 75 seem irrelevant in the line 225. It would be better to cite the reference 36 there.

We have amended this accordingly

Abstract: The wording "Arboviruses cause both seasonal epidemics (e.g. dengue viruses, DENV) and emerging outbreaks (e.g. chikungunya and Zika viruses, CHIKV and ZIKV) with a significant impact on global health." Suggesting contrasting seasonal patterns for the studied viruses" is misleading because seasonality trends have been observed even at the height of CHIKV emergence in the Caribbean (e.g. see Johansson, 2015 Trends Parasitology) and with the prolonged endemicity in the Americas all of the viruses in question exhibit seasonal peaks. So, it's better to remove the viruses' names in the parentheses in the statement above. It's better to refer to refer to Aedes-borne arboviruses instead of just arboviruses text as ZIKV, CHIKV, and DENV are not fully representative of other arboviruses.

We have now revised the abstract and amended the wording as suggested. We now refer to 'Aedes-borne arboviruses' instead of 'arboviruses' throughout the text.

Some typos or tautologies are present throughout the text: "phylogenetic phylogenies" (line 138), "discrete phylogeographic discrete trait analysis" (line 155), "However, inferred dated for these" (line 318), "different different scales" (line 542).

We have now revised the text and amended all grammatical errors accordingly.

Reviewer #3 had no further suggestions

5. Summary and General Comments

No further suggestions
