# Supplementary material for: Convergent trends and spatiotemporal patterns of Aedes-borne arboviruses in Mexico and Central America
Source: PLoS Negl Trop Dis. 2023 Sep 6;17(9):e0011169. doi: 10.1371/journal.pntd.0011169 (PMC10506721; doi:10.1371/journal.pntd.0011169)
Supplement: Attachment — Submitted filename: Response_PNTD-D-23-00195_R2.docx [file pntd.0011169.s022.docx]

**Revised Submission**

**PNTD-D-23-00195R1**

**“Convergent trends and spatiotemporal patterns of arboviruses in Mexico and Central America”**

**Academic Editor: Aparna Krishnavajhala, Elvina Viennet**

Dear Dr. Escalera-Zamudio,

Thank you very much for submitting your manuscript "Convergent trends and spatiotemporal patterns of Aedes-borne arboviruses in Mexico and Central America" for consideration at PLOS Neglected Tropical Diseases. As with all papers reviewed by the journal, your manuscript was reviewed by members of the editorial board and by several independent reviewers. The reviewers appreciated the attention to an important topic. Based on the reviews, we are likely to accept this manuscript for publication, providing that you modify the manuscript according to the review recommendations.

***General response from authors:***

*We thank the editors and reviewers for their positive feedback for this second review process. To enhance the clarity and readability of our study, we have incorporated all changes suggested. Below, we provide a detailed response addressing each issue raised, with line numbers corresponding to the tracked version of the manuscript.*

*We believe that our manuscript is now ready for publication and kindly request your further consideration.*

*Yours faithfully,*

*Marina Escalera Zamudio, Ph.D.*

*Leverhulme Trust ECR fellow*

*Department of Biology*

*University of Oxford*

*Oxford, United Kingdom*

[*marina.escalerazamudio@biology.ox.ac.uk*](mailto:marina.escalerazamudio@biology.ox.ac.uk)

**RESPONSE TO REVIEWERS**

1. **Methods**

Reviewer #2 had no further suggestions

Reviewer #4:

Line 90 I see that Zika was considered in the analysis, but I do not see an explanation for why DENV-3 and DENV-4 weren’t.

Line 131 Sequencing was also obtained from public databases. I am assuming that the sequencing method used was like the authors. Can this be clarified?

*We now provide further information on why DENV-3 and -4 were not considered for this study: "Similar to neighbouring countries, DENV-1 and DENV-2 have prevailed in Mexico since the early 2000s [74,75]. Consequently, historical samples for other DENV serotypes (specifically DENV-3 and DENV-4) are scarce, further limiting the possibility of performing in-depth genomic epidemiology-based analyses. Thus, DENV-3 and DENV-4 samples were excluded." (corresponding to* ***L90****, now* ***L99-103****). This is information is further supported by* ***Supplementary Text 1. Retrospective on Aedes-borne arbovirus epidemics in the Americas and epidemiological surveillance in Mexico****” and* ***Fig 3. Epidemiological trends of DENV in Mexico from 2013 to 2019****, in which the historic predominance of DENV-1 and DENV-2 over other DENV serotypes is evident.*

*Regarding the comment on L131, the sequencing methods employed to generate viral genomes deposited in public databases such as GenBank (distinct from GISAID in this context), is often undisclosed or unavailable. This information is particularly absent for historical records, used in this retrospective study. Moreover, it is unlikely that sequences from before 2016 were generated using the MinION device, as for our viral genomes. Despite representing an important methodological innovation (providing a cost-effective and portable strategy for sequencing), this technology is not widely used as the sequencing standard. In any case, we find it unnecessary to state this in the main text, as all consensus genome sequences utilized adhere to a quality control threshold, mitigating potential biases that could potentially impact our analyses.*

2. **Results**

Reviewer #2 had no further suggestions

Reviewer #4:

Line 213 Discussion about the discrepancy between genomic and epidemiological data could move to the discussion section and expanded upon.

*Related to L213 (now* ***L229****) “Overall, the number of CHIKV genomes per country does not correlate with the cumulative number of cases reported over time (****Supplementary Information,*** ***Fig S3****), indicating a poor association between virus genome and epidemiological data”: we consider this sentence should remain within the results section, as such conclusions were drawn from our exploratory data analyses (****Supplementary Information,*** ***Fig S3****). However, related to this statement, we have now added an additional paragraph in the discussion section:*

*“As evidenced for CHIKV, the cumulative number of virus genomes by country does not correlate with officially reported case numbers, and this is likely to mirror data for other Aedes-borne arboviruses in the Americas. Heterogeneous data sampling prevents a comprehensive outbreak characterization, further accentuating the need to strengthen genomic epidemiology-based surveillance across regions. This also denotes regional disparities in the intensity of virus genome sampling and sequencing through time. As an example of this, for some countries like Brazil, an extensive sampling of ZIKV enabled exploring the spatial and temporal dynamics of the virus across the nation [6], whereas the lack of data from neighbouring countries left substantial knowledge gaps in the global overview of the epidemic within the Americas. Despite a limited virus sequencing for Aedes-borne arboviruses in Mexico, we were still able to successfully quantify and characterize lineage-specific transmission chains across the country. Nevertheless, a more uniform sampling could uncover additional clades, offer finer insights into the geographic distribution of such clades, and enhance viral spread reconstructions at a higher resolution”. (****L660-674****).*

Line 226 seems like a repetition. This was already mentioned in line 29-30

*The line has been removed to start the paragraph with the succeeding sentence (now* ***L244-246****): “The first CHIKV cases were reported in the Eastern Caribbean (also named the Lesser Antilles (Supplementary Information, Table S2), likely leading to larger outbreaks posteriorly observed in other Central Caribbean islands”.*

Line 319 was there any attempt to control for the sampling biases like with a sensitivity analysis?

*This issue addresses the "over-representation of sequences from the USA and Nicaragua" as potential sampling biases that may impact inferred source locations in phylogeographic analyses. In this context, statistical weaknesses relate to dataset robustness and representation. However, “controlling for the sampling biases with sensitivity analyses” can only be done when subsampling has taken place, as in testing multiple datasets subsampled under different strategies. Given the size of our datasets, it was preferable to employ all available sequences rather than subsampling to reduce phylogenetic signal. However, quality control measures implemented included validating inferred phylogenetic trees by comparing general evolutionary trends with publicly available phylogenies. This way, considerable lineage under-sampling represented by long branches can also be identified (see Lemey et al. 2009. https://doi.org/10.1371/journal.pcbi.1000520). Potential biases likely to impact results should also be thoroughly discussed, as has been done in our study (see* ***L641-659****).*

*This has now been clarified in the Methods section ‘Phylogenetic analyses’ as follows: "our final datasets include a total of 420 CHIKV, 375 DENV-1, and 643 DENV-2 genome sequences, selected to represent all countries in North and Central America with virus genome representation sampled though time. Given the reduced size of our final datasets, it was preferable to employ all available sequences rather than subsampling and risking phylogenetic signal. However, quality control measures implemented included validating inferred phylogenetic trees by comparing general evolutionary trends with publicly accessible phylogenies, in order to detect inconsistencies in known divergence patterns both at a broader and deeper node levels." (before* ***L129****, now* ***L144-149****).*

Line 432 what are the minority of the serotyped cases? DENV-3 and DENV-4? It would be helpful to state this.

*See response to the main comment raised for the ‘Methods’ section. L432 (now* ***L457****) has been clarified as follows: “Samples classified as ‘other’ DENV serotypes (neither DENV-1 or DENV-2, mainly corresponding to DENV-4 and 4) comprised the minority of the total serotyped cases”. As a note, the* ***legend for*** ***Figure 3*** *states that “the DENV-3 and DENV-4 serotypes together with potential co-infections (reported between 2013 and 2018) are grouped into the ‘other’ category.*

3. **Conclusions**

Reviewer #2 had no further suggestions

Reviewer #4:

Line 555 what are these high-risk states?

*We have amended* ***L555*** *(now* ***L581-585****) as follows: “However, only an increased targeted surveillance in high-risk states (comprising Chiapas, Oaxaca, Guerrero, Veracruz, Tamaulipas, Quintana Roo, Campeche and Yucatán, denoting those historically most affected by Aedes-borne viruses) could shed light on the cryptic epidemiological behaviour of CHIKV, as well as other emerging and established Aedes-borne arboviruses circulating in Mexico.2. As a note, L63-66 in the Introduction provide an overview of what determines a high-risk state.*

Line 605 seems to suggest vaccine trials among dengue-naïve individuals. However, vaccines like Dengvaxia are only given to people who have previously had dengue and so trials with other vaccines might not be as straightforward.

*We acknowledge the need for further clarification. Thus, we have amended* ***L605*** *(now* ***L637-640****) as “Thus, our results may inform on suitable locations for vaccine trials targeting multiple Aedes-borne arboviruses applicable to naïve individuals, where high numbers of new cases are required to achieve targets of efficacy promptly [90].”, as this corresponds to the vaccine trials we had in mind when structuring the discussion (for details, see corresponding reference).*

4. **Editorial and Data Presentation Modifications**

Reviewer #2 had no further suggestions

Reviewer #4: Minor revision

5. **Summary and General Comments**

Reviewer #2: The issues raised in the first round of review have been addressed and I have no further problems with the manuscript. Congratulations to the authors for this great work!

Reviewer #4: Guiterrez et al. investigated whether there were convergent trends of arboviral evolutionary and epidemiological dynamics in Mexico. Their study also adds to a growing literature on the spatiotemporal patterns of DENV in Southern America.

Abstract and Introduction:

Southwest region includes which parts of Mexico and what is the difference between the southern and southwestern states (line 60 and line 349). It would be helpful to reference how the regions were categorized.

*We acknowledge the need for further clarification. Mexico's regions are geographic zones that group the federal entities of the United Mexican States. While the number and composition of these regions may fluctuate, the eight-region model is a prevailing standard (see* [*https://es.wikipedia.org/wiki/Regiones_de_M%C3%A9xico*](https://es.wikipedia.org/wiki/Regiones_de_M%C3%A9xico)*). This is now referenced in* ***L190*** *in the Methods section.*

Line 17 comes as a surprise. While targeting populations based on age could hold promise, there is no justification in the introduction prior to this statement.

*We have amended* ***L17*** *as follows: “Moreover, mapping arboviral emergence and spread within specific regions can inform on the development of efficient, spatially targeted interventions, including widening genomic-epidemiology surveillance efforts, local campaigns for vector control/clearance, and vaccination campaigns.”*

Line 22, I would suggest citing literature confirming the recent introduction of zika and chikungunya. Or you could move this discussion to the next paragraph which addresses the history of chikungunya.

*We consider this a misunderstanding of the statement given the prior wording used. In order to the improve clarity, we have now amended* ***L22-26*** *as follows: “Highlighted by the more recent introduction of the Zika (ZIKV) and chikungunya (CHIKV) mosquito-borne viruses into the Americas (compared to DENV), contrasting the evolutionary and epidemiological dynamics of emerging and established viruses that have circulated endemically over multiple decades within the same region, could help identify recurrent patterns in Aedes-borne arboviruses sharing ecological niches.” We hope this addresses the issue.*
